# Hypertension and renal impairment in patients with cystinuria: findings from a specialist cystinuria centre

**DOI:** 10.1007/s00240-019-01110-8

**Published:** 2019-02-25

**Authors:** Francesca Kum, Kathie Wong, David Game, Matthew Bultitude, Kay Thomas

**Affiliations:** 1 Department of Urology, Guy’s Hospital, 1st Floor Southwark Wing, Great Maze Pond, London, SE1 9RT UK; 2 Department of Nephrology, Guy’s Hospital, 6th Floor Borough Wing, Great Maze Pond, London, SE1 9RT UK; 3 King’s College London Medical School, Guy’s Campus, Great Maze Pond, London, SE1 1UL UK

**Keywords:** Cystinuria, Hypertension, Chronic kidney disease, Renal impairment, Urolithiasis

## Abstract

Higher blood pressures (mean systolic difference 16.8 mmHg) when compared to matched individuals are already reported in patients with calcium urolithiasis. We present the prevalence of hypertension and renal impairment in patients with cystinuria from our specialist single centre. We analysed our prospective database of adult patients with cystinuria who attend our cystinuria service. This included details of the medical and operative management of their disease. Descriptive statistics were used to analyse and present the data. 120 patients were included with a median age of 40 (19–76) years, 66 were male (55%) and 54 were female (45%). 54/120 patients (45%) were taking medications to prevent stone formation. 78% (94/120) patients reported having undergone one or more stone-related procedure. 59% (55/94) of these having required at least one PCNL or open procedure during their lifetime. Prevalence of hypertension was 50.8% (61/120), and double in males compared to females (62.1% vs. 37.0%, *P* = 0.0063). Mean baseline creatinine was 88.2 (49–153) µmol/l and eGFR was 77.6 (32–127) ml/min/1.73 m^2^. When categorized by CKD stage, only 24.6% (27% vs. 21%, M vs. F) patients had normal renal function (being an eGFR > 89 ml/min/1.73 m^2^). 57.6% patients were CKD stage 2 and 17.8% CKD stage 3. Females had a slightly greater incidence of renal impairment. All patients who have previously undergone a nephrectomy (*n* = 10) or have a poorly functioning kidney (*n* = 19) have renal impairment (CKD stage 2 or 3). Incidence of hypertension in patients with cystinuria is 51%, with a male preponderance. Only 25% of patients with cystinuria have normal renal function. This highlights the long-term cardiovascular and renal risks that the metabolic effects of cystinuria pose, in addition to the challenges of managing recurrent urolithiasis in a young population.

## Introduction

Cystinuria is a genetic disorder, which results in dysfunctional transport of cystine and three dibasic amino acids, namely; ornithine, arginine and lysine [1]. Failure to reabsorb these amino acids from the proximal convoluted tubule results in high urinary concentrations, with subsequent precipitation of cystine as renal calculi. The two key gene mutations which are thought to be responsible for the disorder are SLC3A1 and SLC7A9 [2, 3]. This rare condition affects around 1:2000 people in UK, presenting with recurrent urolithiasis and long-term renal complications [4]. Cystinuria accounts for around 1% of urolithiasis in adults, but 6% in children. Previous research reports varying ages at which patients present with their first calculus; most studies report a young age, with peak age of presentation with their first urinary calculus being between 11 and 20 years of age and estimated 25% develop their first calculus prior to the age of 10 years [4]. The lifelong chronicity of cystinuria, therefore, needs to be managed to preserve renal function [5].

Both hypertension and renal impairment are established cardiovascular risk factors and carry a significant morbidity. In the normal adult population in UK, the background incidence of hypertension, defined as blood pressure ≥ 140/90 mmHg is 31% in men and 27% in women, and increases to 50% in those over 60 years old [6]. When compared to age, sex and ethnic-matched individuals, patients with calcium urolithiasis were found to have significantly higher blood pressures, with a mean systolic difference of 16.8 mmHg [7]. However, these patients are also reported as producing greater amounts of calcium and sodium excretion. Various studies of patients with calcium urolithiasis report stone formation to be associated with a decline in renal function [8–11]. The pathophysiology of hypertension and impaired renal function are closely associated. Patients with renal impairment have shown a disruption in acid–base homeostasis, salt and water handling, which in turn drives hypertension [10]. Similarly, effects of raised arterial blood pressure can directly damage renal parenchyma and vasculature, thus resulting in renal impairment. Patients may progress to end-stage kidney disease (ESRD) requiring renal replacement therapy and amongst other factors, an excessive stone burden can also necessitate nephrectomy.

Few studies have specifically researched the co-morbidities associated with cystinuria and the further long-term complications that ensue from recurrent stone formation. We present the prevalence of hypertension and renal impairment in a large single-centre cohort of cystinuric patients who are managed in a standardised specialist clinic.

## Materials and methods

A prospective database of all patients who attend a specialist cystinuria service at our institution was analysed. All patients seen at this specialist service undergo a standardised clinic review and follow-up protocol, which includes bedside urinalysis; blood pressure measurement using an automatic device; height and weight measurement; and medications review at every appointment. Data collection was retrospective, sourcing information from direct notes review of electronic records, correspondence and operation notes to include basic demographics, diagnosis of hypertension, indicators of renal function (serum creatinine, estimated glomerular filtration rate (eGFR), incidence of a poorly functioning kidney or previous nephrectomy), urinary amino acid levels, management and number of operative interventions.

Presence of hypertension was defined as blood pressure ≥ 140/90 mmHg on two or more clinical readings, and/or documentation of a diagnosis in the clinical notes. If the first reading is elevated, it is repeated after a period of rest in the clinic. If it is still elevated, measurements were then repeated manually by the Nephrologist during the consultation. Patients are referred to their primary care physician if a new diagnosis of hypertension is suspected and recommended for home monitoring. In equivocal cases, 24 h ambulatory blood pressure monitoring is advised. In our clinic the decision to initiate or alter antihypertensive medications is made by the Nephrologist.

Chronic kidney disease (CKD) stage was recorded as per the Kidney Disease Outcomes Quality Initiative guidelines for classification, which uses the Modification of Diet in Renal Disease formula to derive eGFR [12]. Our institution laboratory uses isotope dilution mass spectrometry for measurement of serum creatinine. The most recent creatinine reading from a routine clinical visit was used to derive the eGFR, i.e. not at the time of acute ureteric colic or an intervention.

Descriptive statistics were used to analyse and present the data. Unpaired t-tests were used for continuous data and a *P* value < 0.05 was considered as reaching statistical significance. Pearson’s-*r* correlation coefficient was used to assess linear correlation. Microsoft Excel (Microsoft® Excel® 2016 MSO) and GraphPad (GraphPad Software, Inc. 2017, California) were used for statistical analysis. All data were handled confidentially and anonymously.

## Results

A total of 126 adult patients regularly attend the specialist cystinuria service at our tertiary referral centre. Six patients were excluded due to incomplete data; leaving 120 patients. The patient group included 66 males (55%) and 54 females (45%), with a median age of 40 years (range 19–76 years). 8/120 (7%) were diabetic.

All patients received tailored advice about fluid intake, dietary modifications and weight loss. At the time of analysis, 54/120 patients (45%) were taking regular medications to prevent stone formation (e.g. potassium-citrate, penicillamine, tiopronin, captopril, sodium bicarbonate). A further four patients had previously been taking medications, but stopped due to side effects and poor tolerability. Patients attending the service routinely have their blood pressure and renal function checked at each follow-up episode, in addition to urinalysis for urinary pH, body weight measurements and relevant imaging to assess stone burden.

### Hypertension

**Fig. 1:**
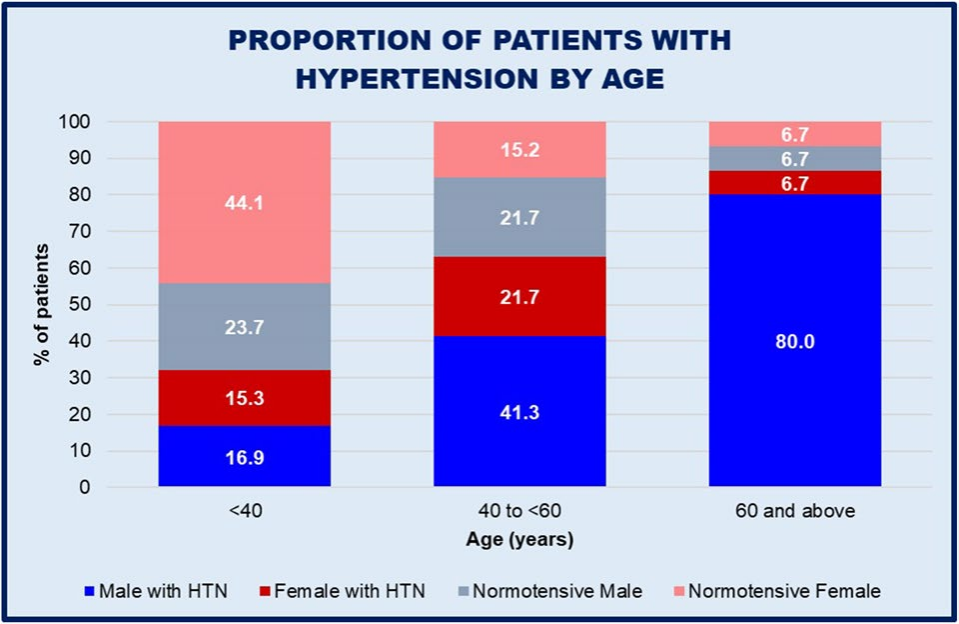
Proportion of patients with hypertension, classified by age and gender

Overall prevalence of hypertension was 50.8% (61/120). Presence of hypertension in males was almost double that of females (62.1% vs. 37.0%, M vs. F, *P* = 0.0063). Patients with hypertension had a significantly greater median age of 48 years, compared with 33 years in those who were normotensive (*P* < 0.0001); with an increasing proportion of males in each progressive age category (Fig. 1). There were no differences in the number of patients who were hypertensive regardless of whether they were taking preventative cystinuria medications or not (26/54, 48% vs. 35/66, 53%. *P* = 0.60).

Of the 61 hypertensive patients, 50 were reported to be taking regular antihypertensive medication. Of the 10 patients who were not taking anti-hypertensives, 3 were under nephrology review for a new diagnosis of hypertension and initiation of treatment, 7 were trying lifestyle interventions for hypertension, and 1 of those patients had declined antihypertensive medications. 30 Patients were taking a single anti-hypertensive; 16 a combination of two agents, 4 patients were taking three anti-hypertensives. One patient was taking captopril as a third line agent for cystinuria, but not for treatment of their hypertension. The types of anti-hypertensive medications prescribed are described in Table 1.

**Table 1:** Anti-hypertensive agents used

Anti-hypertensive class	Number of patients
Angiotensin-converting enzyme inhibitor (ramipril, captopril, lisinopril, perindopril)	30
Angiotensin receptor blocker (valsartan, losartan, candesartan, irbesartan)	11
Beta-blocker (bisoprolol, atenolol)	10
Calcium channel blocker (Amlodipine)	19
Diuretic (furosemide, bendroflumethiazide, indapamide)	3

### Renal function

**Fig. 2:**
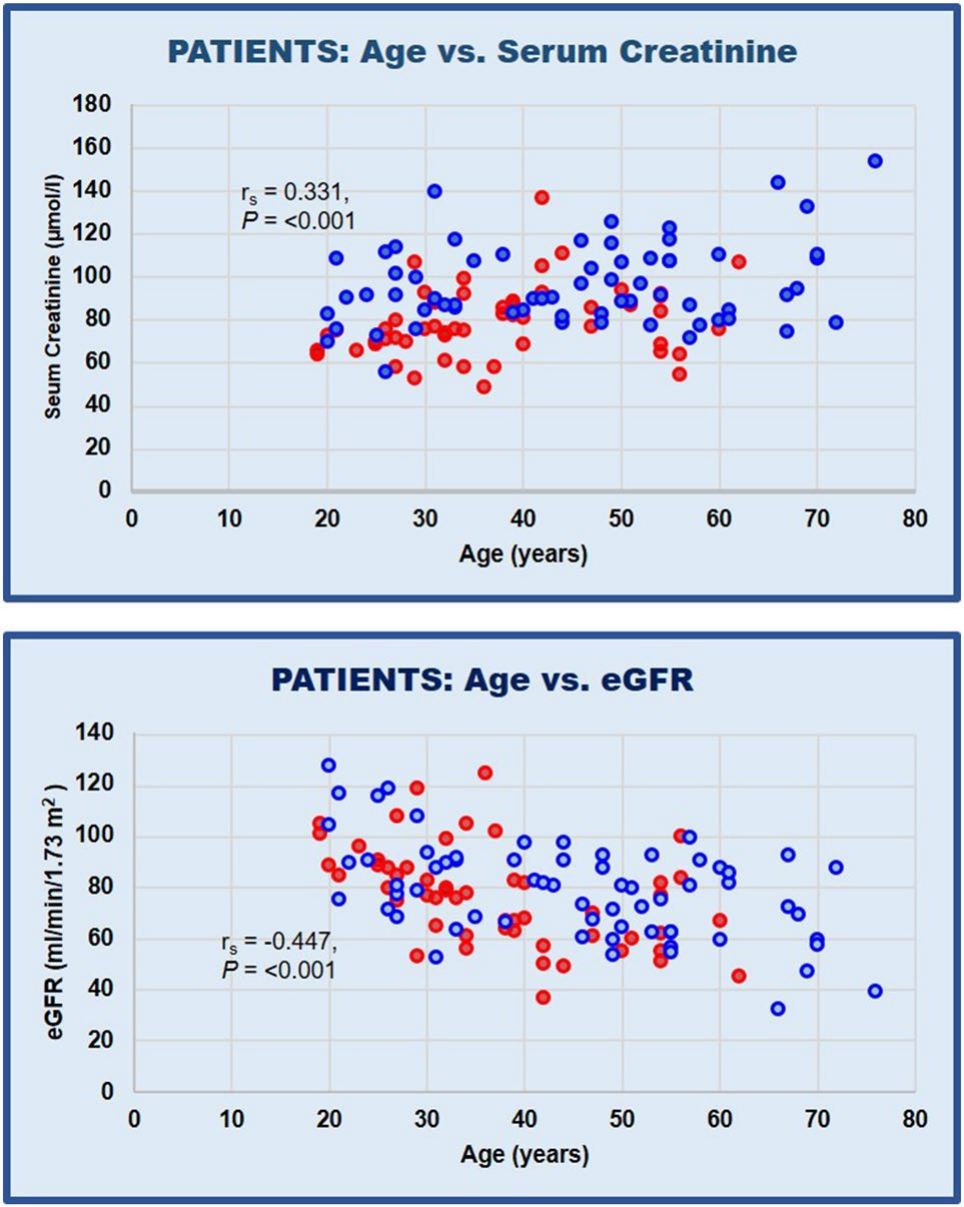
Relationships between **a** patient age and serum creatinine; **b** age and eGFR in female and male patients. Female = , Male =

Mean creatinine was 88.2 µmol/l (range 49–153 µmol/l) and eGFR was 77.6 ml/min/1.73 m^2^ (range 32–127 ml/min/1.73 m^2^). Serum creatinine significantly increases with advancing age (*r*_s_ = 0.331, *P* = < 0.001) (Fig. 2a). Similarly, the relationship between patient age and eGFR is negatively correlated (*r*_s_ = − 0.447, *P* = < 0.001) (Fig. 2b). Age of patients with hypertension were significantly greater, as discussed above.

**Fig. 3:**
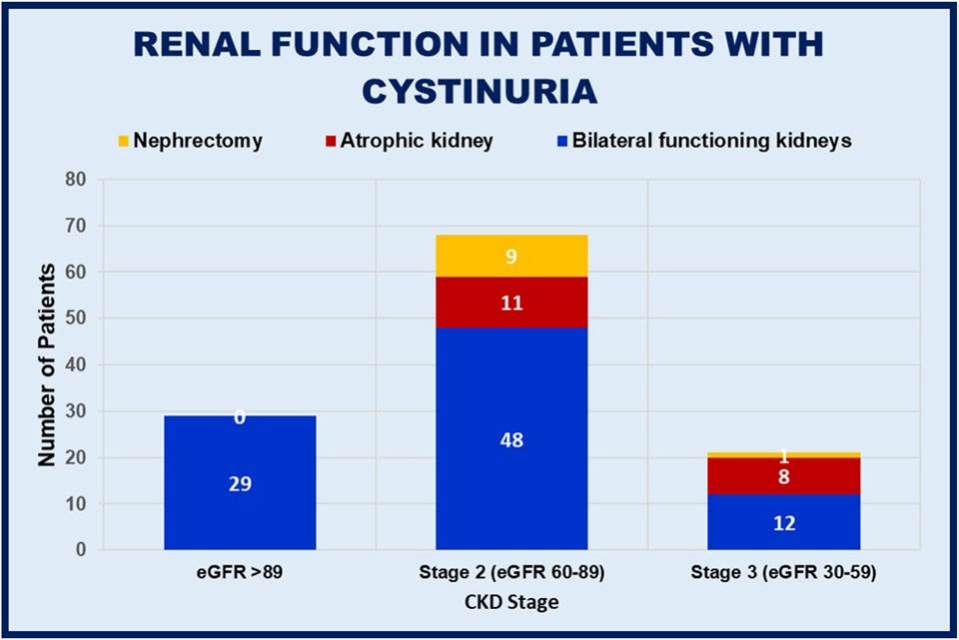
Distribution of CKD stage in patients with cystinuria in relation to previous nephrectomy or known poorly functioning or non-functioning kidney

When categorized by CKD stage, only 24.6% (29/118, 27% vs. 21%, M vs. F) patients had normal renal function (eGFR > 89 ml/min/1.73 m^2^) (Fig. 3). 57.6% patients (68/118, 56% vs. 60%, M vs. F) were CKD stage 2 (eGFR 60–89 ml/min/1.73 m^2^) and 17.8% (21/118, 17% vs. 19%, M vs. F) were CKD stage 3 (eGFR 30–59 ml/min/1.73 m^2^). No patients had CKD stage 4 or 5 (eGFR 15–29 ml/min/1.73 m^2^ or < 15 ml/min/1.73 m^2^, respectively), or ESRD and renal transplant. Females in our study have a slightly greater prevalence of renal impairment than males, which may advocate closer monitoring for renal loss in females. This is somewhat unexpected given that the prevalence of hypertension is significantly greater in males than females in both our study and the background healthy population.

All patients who have previously undergone a nephrectomy (*n* = 10, 8.3%) or have a poorly functioning kidney (*n* = 19, 15.8%) have renal impairment (CKD stage 2 or 3). The definition of a poorly functioning, atrophic or non-functioning kidney was taken from functional DMSA studies or ultrasonography. 90% of patients with previous nephrectomy had CKD stage 2 and 10% CKD stage 3. There were 61% of patients with a poorly functioning kidney who had CKD stage 2 and the remaining 39% were CKD stage 3. The proportion of patients with hypertension increased per CKD stage. Of the patients with normal renal function, 34.1% (10/29) were hypertensive, 50% (34/68) for CKD stage 2, and 81% (17/21) of those with CKD stage 3.

When considering proteinuria as a marker of renal impairment, to guide target blood pressure for therapy, and use of angiotensin-converting-enzyme inhibitors and angiotensin receptor blockers, 69.1% (83/120) had trace, 1 +, or 2 + protein in their urine (Table 2). There were 10 patients with 2 + proteinuria, of which 7/10 (70%) were hypertensive. The proportions of patients who had hypertension and 2 + proteinuria were not significantly different from those with absence or trace proteinuria (*P* = 0.195).

**Table 2:** Proteinuria on urinalysis and number of patients with hypertension and uPCR

Protein on urine dipstick	*n*	Hypertension	% Hypertensive	Median uPCR (mg protein/mmol creatinine)
Negative	37	13	35.1	9.5
Trace	41	25	61.0	7.5
1+	32	16	50	9
2+	10	7	70	50

Urine protein–creatinine-ratio (uPCR) measurements were obtained for 40 patients (Table 2). It is intended to measure the uPCR in any patient with 1 + proteinuria or more and correct the protein level for the concentration of solute due to the large volumes of fluid the patients are taking. In those measured, mean 19.8 mg protein/mmol creatinine, median 9 mg protein/mmol creatinine, and of these uPCR was found to be elevated (> 15 mg protein/mmol creatinine) in 14 patients (35%).

Patients report to having undergone a greatly varying number of operations throughout their disease course, ranging from 0 to 32 stone-related procedures (median = 3). A ‘stone-related procedure’ included an emergency stenting procedure, nephrostomy tube insertion, ureteroscopy, extracorporeal shockwave lithotripsy (ESWL), percutaneous nephrolithotomy (PCNL) and open procedures. 78% of patients (94/120) had one or more stone-related procedure, with 59% (55/94) of these having required at least one PCNL or open procedure (median = 1, range 1–11 procedures). There were no gender differences in the reported number of procedures overall (3.7 vs. 4.1, M vs. F, *P* = 0.66).

**Fig. 4:**
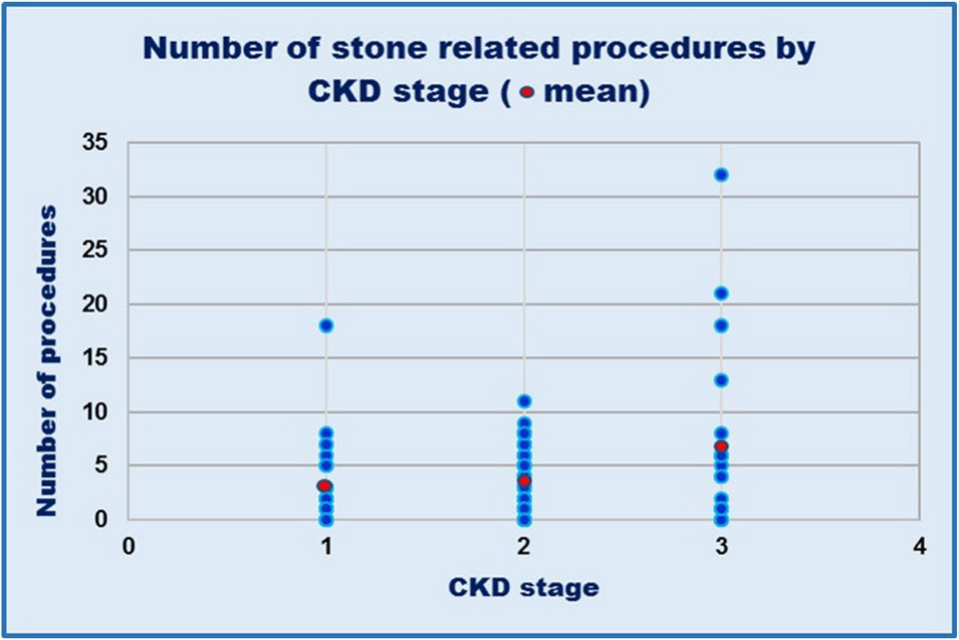
Number of operative interventions required for stones in relationship with CKD stage. Means: CKD stage 1, 3 procedures per patient; CKD stage 2, 3.2 procedures per patient; CKD stage 3, 6.4 procedures per patient

On reviewing the number of operative procedures in relation with renal function, the number of procedures each patient had undergone for their stone disease bore no correlation with eGFR (*r*_s_ = − 0.127, *P* = 0.17). However, when categorised by CKD stage (Fig. 4), although the mean number of procedures undergone were similar for patients with CKD stages 1 or 2 (3.0 and 3.2 procedures per patient, *P* = 0.74), patients with CKD stage 3 had a significantly greater mean number of procedures (6.4 procedures per patient) when compared with CKD stage 1 (*P* = 0.03) and CKD stage 2 (*P* = 0.003).

When considering if the use of preventative cystinuria medications was related to CKD prevalence or stage, there were similar numbers of patients in each group overall, *P* = 0.22. However, a greater number of patients with CKD stage 3 were not taking preventative medications, but there were small numbers in this group (Table 3).

**Table 3:** CKD prevalence classified according to use of cystinuria preventative medications

	CKD stage 1 (*n* = 29)	CKD stage 2 (*n* = 68)	CKD stage 3 (*n* = 21)
Taking medication (*n* = 52)	13 (25%)	32 (62%)	7 (13%)
No medications (*n* = 66)	16 (24%)	36 (55%)	14 (21%)
*P* value	0.440	0.496	0.031

## Discussion

In our study of 120 patients with cystinuria, we found a greater prevalence of hypertension and renal impairment than reported in the general population and calcium stone formers [7, 9, 10]. This is supported by a similar series of over 300 patients with cystinuria from multiple centres throughout France and a smaller study of 76 patients from 2 UK centres [13, 14]. Our study differs as this is the largest cohort of patients who all attend a dedicated clinic in a single centre and are managed by the same multi-disciplinary team with a standardised protocol.

It is already widely accepted that patients with calcium urolithiasis have a greater prevalence of hypertension than the normal population [7, 8, 15, 16]. Worcester et al. reported no significant blood pressure difference between those with cystinuria stones or other stone causes [16]. The prevalence of hypertension in the normal healthy population hypertension is estimated as being is 31% in men and 27% in women [6]. Prevalence of hypertension in our patient group was substantially greater (50.8%) than in the French cohort (28.6%) [13]. However, their criterion for reporting of hypertension was dependent on individual physician diagnosis rather than a standardized cut-off blood pressure reading. Male gender was also significantly associated with hypertension in our cohort (62.1% vs. 37.0%, M vs. F), with incidence of hypertension in males with cystinuria being more than twofold that of their female counterparts. This is not explained by the number of procedures in our series, which were similar between both genders (3.7 vs. 4.1, M vs. F, *P* = 0.66). Hypertension is a potentially modifiable cardiovascular and CKD risk factor; therefore, these findings support the need for adequate treatment and routine screening for hypertension in this patient group, which is already established in our specialist service.

Impaired renal function is more prevalent in patients with urolithiasis than those without stone formation [8–10, 15]. Other series confer a similar proportion of patients with cystinuria having renal impairment, as demonstrated in our series. Both Prot-Bertoye et al. and Rhodes et al. reported a comparable distribution of CKD stage in their cohorts, in which reports of only 22.5% and 30% having normal renal function (CKD stage 1, eGFR > 89 ml/min/1.73 m^2^); vs. 24.5% in our UK series [13, 14]. Prot-Bertoye et al. described 11.1% patients as having eGFR < 30 ml/min/1.73 m^2^, a similar proportion to the 17.8% found in our series [13]. Similarly, Rhodes et al. reported 5% patients having eGFR < 30 ml/min/1.73 m^2^ and three patients (4%) had ESRD with subsequent renal transplant [14]. It is encouraging to report that none of our cystinuric patients have CKD stages 4 or 5 (eGFR < 30 ml/min/1.73 m^2^) or ESRD. The pathophysiological mechanisms which result in impairment of renal function in patients with cystinuria are related to the insolubility of cystine in urine. Stone formation often begins in childhood, cystine crystals accumulate and cause obstruction of the ducts of Bellini along with interstitial fibrosis and glomerulosclerosis, which results in the decline in renal function [17]. However, the frequency of operative procedures, ureteric obstruction and urosepsis may further contribute to the impaired renal function observed.

In the general UK population, the estimated prevalence of CKD stages 3–5, both diagnosed and un-diagnosed, using the 2014 CKD prevalence model is 6.1% (95% CI 5.3–7.0%) [18]. In our cystinuric cohort, the prevalence of patients with CKD stage 3 was almost threefold greater than that of the healthy control population (17.8%), hence indicating the potential health burden and greater morbidity of these patients. Assimos et al. has reported that patients with cystinuria have higher serum creatinine levels compared with calcium urolithiasis patients, which can be aligned with progression of CKD [19].

When considering the type and number of previous surgeries as a potential contributory factor for development of hypertension and renal impairment, there was no significant difference for hypertensive vs. normotensive patients in our cohort. However, when classified by CKD stage, there was a significant correlation between CKD severity stage and overall number of procedures. This provides support for optimizing non-operative management (e.g. diet, fluid intake, weight loss, urinary alkalinisation, and chelating agents) to prevent stone formation and preserve renal function. Gambaro et al. conducted a review of CKD in relation to treatments for urolithiasis and concluded that ESWL and minimally invasive treatments for renal stones in isolation do not negatively affect renal function, as the damage to renal parenchyma though present is small and often recovers, but overall stone burden that requires multiple complex procedures and pre-existing renal impairment may contribute [20]. When considering long-term outcomes, Barbey et al. report reduced creatinine clearance in patients with cystinuria who have undergone a greater number of stone-related procedures [21]. Furthermore, in addition to male gender being independently associated with hypertension, being male was a predisposing factor to requiring nephrectomy in a series of 40 patients with cystinuria [19]. When compared with calcium oxalate stone formers, a greater requirement for surgical stone removal and subsequent progression to nephrectomy was found in those with cystinuria, thus indicating that more invasive operations, PCNL or open procedures should be avoided in these at-risk patients [19].

Limitations of data collection are present in the retrospective collection of some parameters, particularly relating to the accurate recording of the number of operative interventions. The reported number of stone-related interventions each patient had undergone was reliant on patients’ own reporting; operation note review, if the procedure was performed at our institution; or accurate reporting in clinical correspondence if procedures were performed at another centre. Considering that our patient group are mostly referred from their local hospital, several patients had undergone treatment to referral for formal diagnosis or further specialist treatment. Similarly, following diagnosis and in the interim between follow-up episodes, emergency procedures for renal colic, such as ureteric stenting or nephrostomy tube insertion are frequently also performed at the local hospital. In this paper, we assumed that each stone-related procedure, ranging from ureteric stenting to ESWL to PCNL were of equivalent clinical implication. Whilst clearly this is not the case, there is no currently agreed standard weighting for each procedure.

A limitation of our population reported is the lack of an age-matched control group with non-cystine stones; however, we have used published findings of the incidence and hypertension and CKD for comparison. The definition of a poorly functioning kidney was taken from either functional DMSA studies or ultrasonography. All patients have had a renal tract ultrasound, but renograms are not available for all patients, therefore, we acknowledge that this is a heterogenous group. Some patients had a prior nephrectomy, therefore, a DMSA study would provide no further information.

## Conclusions

Prevalence of hypertension in patients with cystinuria is greater than that of the normal population, with a strong male preponderance. Half of all our patients with cystinuria are hypertensive despite being a young group. Many patients with cystinuria have evidence of renal impairment, with less than 25% having a normal eGFR (≥ 90 ml/min/1.73 m^2^). Therefore, emphasising the importance of strategies to both minimise intervention for stones and to reduce risk of CKD progression. This preliminary data highlights the long-term cardiovascular and renal risks that the metabolic effects of cystinuria pose, in addition to the challenges of managing recurrent urolithiasis in a young population. Further investigation into factors which are contributing to the decline in renal function, such as significance of operative interventions and stone burden should be explored. There is also further scope for a clinical risk stratification for this select group of patients.

## Abbreviations

CKD: Chronic kidney disease
eGFR: Estimated glomerular filtration rate
ESRD: End-stage renal disease
ESWL: Extracorporeal shockwave lithotripsy
uPCR: Urine protein–creatinine-ratio
PCNL: Percutaneous nephrolithotomy
